# The Tenofovir Pre-Exposure Prophylaxis Trial in Thailand: Researchers Should Show More Openness in Their Engagement with the Community

**DOI:** 10.1371/journal.pmed.0020346

**Published:** 2005-10-25

**Authors:** Arlene Chua, Nathan Ford, David Wilson, Paul Cawthorne

**Affiliations:** **1**Médecins Sans FrontièresBangkokThailand

Two recent articles in *PLoS Medicine* [1,2] criticise the role played by activists in raising concerns about the tenofovir trial for HIV prophylaxis. We fully support the fact that activism should be based on informed opinion, rather than speculation, unwarranted criticism, overreaction, or sensationalising facts [1], and believe that in Thailand, the concerns raised by activists are entirely legitimate.

The key community groups that have expressed concerns about the tenofovir trial in Thailand are the Thai Drug Users Network (TDN) and the Thai AIDS Treatment Advocacy Group ( TTAG), which are described in Box 1. These community groups, which can justifiably claim to represent Thai drug users, are well informed about the trial, but their objective concerns have been ignored by the trial investigators. Contrary to the assertion of Joep Lange [2] “that the investigators did consult intensely with community groups concerned”, TDN and TTAG were not consulted about the trial design and conduct until a very late stage, after several attempts to engage with the investigators had been rebutted. TDN and TTAG had attempted to constructively engage with the investigators since October 2004; they confined their statements of concern to private letters and meetings with the investigators, until the matter was made public in a *Lancet* editorial in March 2005 [3].

**Box 1. Community Groups Representing IDUs in Thailand**

**Thai Drug Users Network**

TDN, Thailand's only drug users' group, was established in December 2002 in response to the health and human rights crisis facing drug users in Thailand, particularly injectors. TDN's mission is to promote the basic human rights of people who use drugs, in order for them to be able to live with dignity in Thai society. TDN undertakes peer-driven HIV prevention, care, and support for IDUs, has supported the Thai government National Harm Reduction Working Group's activities, and has provided technical input to United Nations Office on Drugs and Crime and World Health Organization consultations. Projects are jointly implemented with other organisations, such as the project Preventing HIV and Increasing Care and Support for IDUs in Thailand, which is funded by the Global Fund to Fight AIDS, Tuberculosis, and Malaria (GFATM).

**Thai AIDS Treatment Action Group**

TTAG was founded in December 2002 by the founding chairman of the Thai Network of People Living with HIV/AIDS (PLHA) in order to promote leadership and advocacy among PLHA. TTAG's mission is to promote equal access to AIDS prevention and treatment for all individuals through policy advocacy and coalition building, and by strengthening the capacity of people living with HIV/AIDS to advocate for their human rights. Projects include Preventing HIV and Increasing Care and Support for IDUs in Thailand, which is funded by GFATM, and the Mekong Region Treatment Preparedness Initiative.

**Key Objections of TDN and TTAG to the Tenofovir Pre-Exposure Prophylaxis Trial in Thailand in Its Present Form**

- Absence of community consultation during the trial design and lack of meaningful consultation during its implementation.
- Best current prophylactic methods unavailable to trial participants.
- No commitment by trial sponsors to promote the safety of trial participants when accessing services.
- No commitment by the researchers to work, after the trial, with the Thai Ministry of Public Health towards price reductions of tenofovir.

**Principle Recommendations**

- Urgently establish a committee, chaired by the US Centers for Disease Control and Prevention, to address key HIV prevention, treatment, and care issues for Bangkok IDUs in the context of the trial. Members should include two TDN representatives, and Thai Red Cross, government, and nongovernmental representatives.
- Involve TDN in trial outreach and education, including curriculum development.
- Develop partnerships to ensure the safety of trial participants when accessing services; for example, the Bangkok Metropolitan Authority should host police training workshops on harm reduction, and TDN should be involved in these activities.
- Commit to supporting TDN in efforts to ensure at least two years of post-trial tenofovir to trial participants, and to working with the Thai Ministry of Public Health towards price reductions.

One major concern about the trial was the failure to provide sterile needles and syringes. Singh and Mills assert that this is consistent with Thai government policy [1]. Long prison terms and death sentences are the norm for drug-related offences [4], and Thai police, who have wide discretionary powers, still occasionally use possession of needles as evidence to arrest suspected drug users. Thus, although needles and syringes are available over the counter from most retail pharmacies, intravenous drug users (IDUs) are afraid to purchase them and, indeed, are often afraid to use services known to be provided for drug users. There is, however, no law or policy forbidding the distribution of clean needles and syringes, and preventing the investigators from doing so.

In fact, the situation in Thailand is improving, with the National Harm Reduction Working Group, chaired by the Ministry of Public Health, taking steps to increase activities in this domain. In 2004, at the 15th International AIDS Conference in Thailand, the prime minister said, “We are now implementing a harm reduction program to reduce the risk of HIV infection among injecting drug users…the program…will be conducted through concerted collaboration among solo UN agencies, government bodies and non-governmental organizations including the Drug User Network” [5].

The reason clean injection materials are not distributed within the trial is because the United States government, who sponsors the trial, bans federally funded organisations (including the Centers for Disease Control and Prevention, who are overseeing the trial) from supporting needle and syringe exchange.

Irrespective of whether a needle exchange exists in Thailand, or what the policies of the trial's funders are regarding needles and syringes, investigators have a duty to respect the Helsinki Declaration requirement that “benefits, risks, burdens and effectiveness of a new method should be tested against those of the best current prophylactic, diagnostic and therapeutic methods” [6].

The HIV/AIDS community in Thailand is not naive about the ethics of clinical trials: many have been directly or indirectly affected by previous AIDS drug trials in Thailand that have raised ethical concerns [7,8]. Nevertheless, TDN and TTAG have, from the beginning, made it clear that they support the development of innovative prevention tools to reduce the burden of global HIV, and would like this trial to go ahead.

We believe that the disagreements surrounding the tenofovir trial in Thailand would have been avoided if the investigators had set out to engage the community more openly, and if the wealth of established knowledge among community members could have contributed enormously to the success of the trial design and implementation. TDN and TTAG have made recommendations (see Box 1) that represent a constructive way for this trial to move forward. Mechanisms that ensure systematic involvement of legitimate representatives of the affected community as partners in research are the only way to ensure that future trials will proceed in a more productive way.
